# NANOG/NANOGP8 Localizes at the Centrosome and is Spatiotemporally Associated with Centriole Maturation

**DOI:** 10.3390/cells9030692

**Published:** 2020-03-11

**Authors:** Erika Mikulenkova, Jakub Neradil, Ondrej Vymazal, Jan Skoda, Renata Veselska

**Affiliations:** 1 Department of Experimental Biology, Faculty of Science, Masaryk University, 61137 Brno, Czech Republic; mikulenkova@mail.muni.cz (E.M.); jneradil@sci.muni.cz (J.N.); ovymazal@mail.muni.cz (O.V.); veselska@sci.muni.cz (R.V.); 2 International Clinical Research Center, St. Anne’s University Hospital, 65691 Brno, Czech Republic; 3 Department of Pediatric Oncology, University Hospital Brno and Faculty of Medicine, Masaryk University, 62500 Brno, Czech Republic

**Keywords:** NANOG, NANOGP8, human, localization, centrosome, mother centriole

## Abstract

NANOG is a transcription factor involved in the regulation of pluripotency and stemness. The functional paralog of NANOG, NANOGP8, differs from NANOG in only three amino acids and exhibits similar reprogramming activity. Given the transcriptional regulatory role played by NANOG, the nuclear localization of NANOG/NANOGP8 has primarily been considered to date. In this study, we investigated the intriguing extranuclear localization of NANOG and demonstrated that a substantial pool of NANOG/NANOGP8 is localized at the centrosome. Using double immunofluorescence, the colocalization of NANOG protein with pericentrin was identified by two independent anti-NANOG antibodies among 11 tumor and non-tumor cell lines. The validity of these observations was confirmed by transient expression of GFP-tagged NANOG, which also colocalized with pericentrin. Mass spectrometry of the anti-NANOG immunoprecipitated samples verified the antibody specificity and revealed the expression of both NANOG and NANOGP8, which was further confirmed by real-time PCR. Using cell fractionation, we show that a considerable amount of NANOG protein is present in the cytoplasm of RD and NTERA-2 cells. Importantly, cytoplasmic NANOG was unevenly distributed at the centrosome pair during the cell cycle and colocalized with the distal region of the mother centriole, and its presence was markedly associated with centriole maturation. Along with the finding that the centrosomal localization of NANOG/NANOGP8 was detected in various tumor and non-tumor cell types, these results provide the first evidence suggesting a common centrosome-specific role of NANOG.

## 1. Introduction

NANOG is a homeodomain transcription factor involved in embryonic stem cell (ESC) self-renewal and pluripotency [1,2,3]. NANOG has been identified as a common stem cell marker and is crucial for the regulation of the cancer stem cell phenotype in several types of solid tumors [4,5,6,7,8,9,10]. Together with SOX2 and OCT4, NANOG plays a key role in maintaining the features of ESCs [11,12,13]. NANOG is highly expressed in pluripotent cells, such as ESCs and embryonal carcinoma cells, and its expression is downregulated upon differentiation [1,14].

Human NANOG is encoded by the *NANOG* gene, which is located in chromosomal region 12p13.31 [15]. Two NANOG isoforms, NANOG and NANOG-delta 48, resulting from alternative splicing [15], and 11 pseudogenes, NANOGP1 to NANOGP11, have been described in humans [16]. Based on the NCBI protein database, while the human NANOG protein (NP_079141.2) consists of 305 amino acids, the NANOG-delta 48 isoform (NP_001284627.1) lacks amino acids 167–182. The *NANOGP8* pseudogene represents a transcribed retrogene that has 99% homology with NANOG. Thus, *NANOGP8* can potentially code for a 305 amino acid protein (NP_001342210.1) that differs from NANOG by only three amino acids. A study focused on the expression of NANOG paralogs found that human ESCs express large amounts of NANOG [17]. In contrast, most human cancer cells express NANOGP8 [18], although its expression is not restricted solely to transformed cells [17,18,19].

NANOG is a homeobox-containing protein that is typically localized in the cell nucleus [20,21]. However, the cytoplasmic localization of this protein has also been described [22,23], even though the role of cytoplasmic NANOG has not been fully elucidated. During our ongoing study on rhabdomyosarcoma, we unexpectedly noticed an atypical cytoplasmic localization of NANOG, which resembled the perinuclear localization of centrosomes. Given these surprising results, we sought to examine NANOG protein localization across a panel of various tumor and non-tumor cell types. In this report, we present our comprehensive analysis of this phenomenon and provide the first evidence for an intriguing centrosomal localization of NANOG/NANOGP8, which was detected as common among several cell types.

## 2. Materials and Methods

### 2.1. Cell Lines and Cell Culture

Nine tumor cell lines of different origins and two non-tumor cell lines were used in this study; a brief description of these cell lines is provided in Table 1. NSTS-34 and NSTS-35 tumor samples were obtained from patients undergoing rhabdomyosarcoma resection surgery. Written informed consent was obtained from each patient or patient’s legal guardian prior to participation in this study. The study was conducted in compliance with the Declaration of Helsinki, and the study protocol (#12/Si/2011) was approved by the Research Ethics Committee of the School of Science (Masaryk University). The paraformaldehyde-fixed CCTL14 human embryonal stem cells were a gift from Dr. Hampl [24]. RD and NTERA-2 cells were cultured in high glucose DMEM supplemented with 10% fetal calf serum (FCS), NSTS-11, NSTS-34, NSTS-35, GM7, HGG-02, and KF1 cells were maintained in DMEM with 20% FCS, Daoy cells in DMEM with 10% FCS, and SH-SY5Y cells were cultured in DMEM/Ham’s F12 medium supplemented with 20% FCS. All media were supplemented with 2 mM glutamine, 100 IU/mL penicillin, and 100 μg/mL streptomycin; the addition of 1% non-essential amino acids (all from Biosera, Nuaillé, France) was used for RD, SH-SY5Y, and Daoy culture media. Cells were maintained at 37 °C in a humidified atmosphere containing 5% CO_2_.

### 2.2. Immunofluorescence

For indirect immunofluorescence (IF), cells were cultivated on coverslips in Petri dishes for 1–2 days (grown to 80–90% confluence), depending on the cell proliferation rate. IF was performed as previously described [29]. The primary and secondary antibodies used in these experiments are listed in Table 2. Anti-CP110 antibody was a gift from Dr. Cajanek [30]. Negative controls were prepared by omitting the primary antibody. After a final wash with PBS, the coverslips were mounted using ProLong^TM^ Diamond Antifade Mountant (Thermo Fisher Scientific, Waltham, MA, USA). An Olympus BX-51 microscope was used for sample evaluation; micrographs were captured using an Olympus DP72 CCD camera and analyzed using the Cell^P imaging system (Olympus, Tokyo, Japan).

### 2.3. Western Blotting

Fifty micrograms of whole-cell extracts were loaded onto 10% sodium dodecyl sulfate (SDS)-polyacrylamide gels, electrophoresed, and blotted onto polyvinylidene difluoride membranes (Bio-Rad Laboratories GmbH, Feldkirchen, Germany). The membranes were blocked with 5% nonfat milk in PBS with 0.05% Tween 20 (PBS-Tween) and then incubated with primary antibody diluted in blocking solution at 4 °C overnight. After rinsing with PBS-Tween, the membranes were incubated with the corresponding secondary antibody at room temperature for 60 min. After rinsing with PBS-Tween, chemiluminescent detection using Amersham^TM^ ECL^TM^ Prime Western Blotting Detection Reagent (GE Healthcare, Little Chalfont, UK) was performed according to the manufacturer’s instructions. To analyze the nuclear and cytoplasmic fractions separately, a Nuclear Protein Extraction kit (Thermo Fisher Scientific) was used according to the manufacturer’s instructions. Forty-five microliters of protein extract was loaded onto 10% (SDS)-polyacrylamide gels. The primary and secondary antibodies used in the experiments are listed in Table 2. Anti-Lamin B2 and anti-α-tubulin antibodies served as the controls for the purity of the nuclear and cytoplasmic cell fractions, respectively. Three biological replicates were analyzed for each sample.

### 2.4. Transient Transfection

RD and NTERA-2 cells were transiently transfected with 2 µg of pCMV6-AC-GFP vector encoding NANOG (OriGene, Rockville, MD, USA). The cells were cultured on coverslips in Petri dishes with complete growth medium overnight and then transfected using TurboFectinTM 8.0 (OriGene) according to the manufacturer’s protocol. The transient transfection experiments were repeated twice.

### 2.5. Immunoprecipitation and Protein Digestion

Cells were overlaid with lysis buffer (10 mM Tris-HCl pH 7.9, 420 mM NaCl, 0.1% NP-40) with the addition of a protease inhibitor cocktail (Sigma-Aldrich, St. Louis, MO, USA). Cell lysates were frozen and thawed three times and then sonicated 12 times (1 s on/2 s off) with 50% amplitude. Lysates were centrifuged, and supernatants were incubated with Ab precoated Dynabeads^TM^ protein G beads (Thermo Fisher Scientific) prepared according to the manufacturer’s protocol at room temperature for 60 min. Beads were washed with washing buffer (50 mM Tris-HCl pH 7.5, 150 mM NaCl) and transferred into clean tubes. Bound protein elution was performed by adding 100 µl of 8 M urea followed by 15 min incubation. Elution was repeated two times, and yields were combined.

Urea-eluted samples were loaded on a 10 kDa Amicon^®^ MWCO filter and processed by the FASP method [31]. Briefly, proteins were washed with 8 M urea followed by centrifugation. The reduction of the disulfide bonds was performed with 10 mM dithiothreitol at room temperature for 15 min. Acetylation was performed with 50 mM iodoacetamide at room temperature for 15 min. After washing with 25 mM triethylammonium bicarbonate, Pierce^TM^ Trypsin Protease (Thermo Fisher Scientific) was added at a 1:50 ratio, and the digestion proceeded overnight at 37 °C.

### 2.6. Mass Spectrometry and Data Processing

LC-MS/MS analysis of tryptic peptides was performed using a Dionex UltiMate 3000 RSLCnano liquid chromatograph connected to a micrOTOF-Q II mass spectrometer (Bruker, Billerica, MA, USA). Samples were separated on a C18 Acclaim PepmapRSLC separation column (25 cm, I.D. 75 µm, particles 2 µm) using a flow rate of 300 nl/min of solvent A (0.1% formic acid) and solvent B (0.1% formic acid in 20/80 H_2_O/ACN (vol/vol)) mixed in a 90 min-long linear gradient from 4% to 55% of solvent B. The mass spectrometer was operated at a scanning frequency of 4 Hz and in a data-dependent mode. The five most intensive precursor ions were fragmented using CID fragmentation using an isolation width of 1.2 Th. The collision energy was adjusted between 27 and 48 eV as a function of the m/z value. Dynamic exclusion of the fragmented precursor was enabled for 30 s.

Raw LC-MS/MS data were converted into mzML format using ProteoWizard [32] and further processed by the Crux pipeline [33]. MS/MS spectra identification was performed by the Tide search engine using the *Homo sapiens* protein fasta file as a database (SwissProt sequences obtained from UniProt protein database), 40 ppm and 0.05 Da as parent and fragment tolerances (respectively) and oxidation and carbamidomethylation as potential and fixed modifications (respectively). Percolator [34] was then employed to validate spectral matches, perform protein inference, and calculate false discovery rates (FDR). Only those proteins passing 5% FDR were retained. FlashLFQ [35] was used for chromatographic peak area estimation using 10 ppm precursor tolerance and list of identified peptide features as input. For each protein, its peptide areas were combined into single values by averaging the areas of the three most intensive peptides (TOP3 approach) [36].

### 2.7. RT-qPCR

Total RNA was extracted using the GenElute^TM^ Mammalian Total RNA Miniprep kit (Sigma-Aldrich), and RNA concentration and purity were determined spectrophotometrically. For all samples, equal amounts of RNA were reverse transcribed into cDNA using M-MLV reverse transcriptase (Top-Bio, Prague, Czech Republic) and oligo-dT priming (Qiagen Inc., Valencia, CA, USA). Quantitative PCR was performed in 10 µL reaction volumes using the KAPA SYBR^®^ FAST qPCR Kit (Kapa Biosystems, Wilmington, MA, USA) and analyzed using the 7500 Fast Real-Time PCR System and 7500 Software v. 2.0.6 (both Life Technologies, Carlsbad, CA, USA). Technical triplicates were analyzed for each of the three biological replicates and relative gene expression levels were determined using the 2^−∆∆CT^ method [37]. The housekeeping gene *HSP90AB1* was used as an endogenous reference control. The primer sequences used in this study are summarized in Table 3. NANOG primers were designed as previously published [38].

## 3. Results

### 3.1. Centrosomal Localization of the NANOG Protein

**Figure 1:**
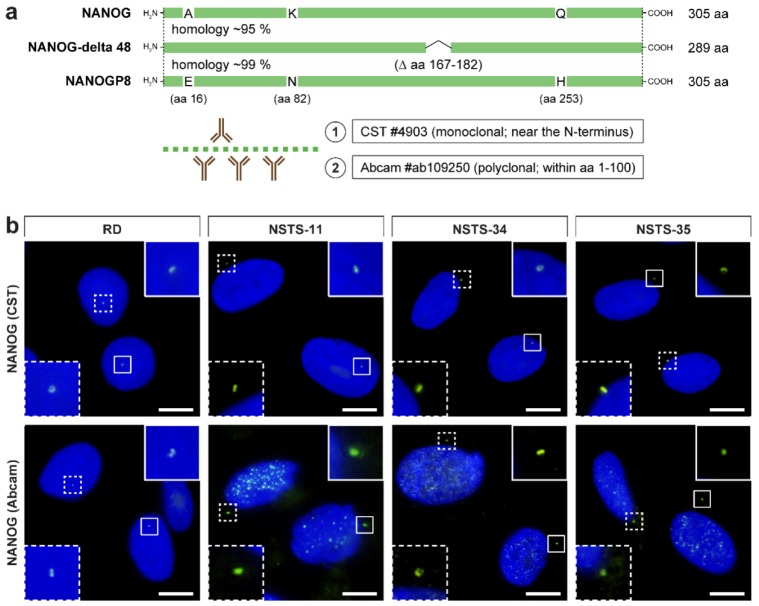
Overview of epitopes of the anti-NANOG antibodies used in this study and their detection of the perinuclear localization of NANOG in rhabdomyosarcoma cell lines. (**a**) Schematic illustration of NANOG isoforms and the NANOGP8 paralog and overview of epitopes of the anti-NANOG antibodies used in the study: (1) #4903, Cell Signaling Technology (CST) and (2) #ab109250, Abcam. Note the marked homology of the NANOG and NANOGP8 protein sequences in the protein region, which served as the immunogen for producing the anti-NANOG antibodies. (**b**) NANOG protein (green) was detected in the cytoplasm near the cell nucleus in the form of one or two punctae using two anti-NANOG commercial antibodies (#4903, Cell Signaling Technology (CST) and #ab109250, Abcam). The nuclei were counterstained with Hoechst 33342 (blue). For each image, regions of interest and the respective close-ups are indicated by the dashed and solid boxes. Scale bars, 10 μm.

During our ongoing study aimed at the analysis of established and patient-derived rhabdomyosarcoma cell lines, we revealed an intriguing extranuclear localization of NANOG protein. Using anti-NANOG commercial antibody (#4903, Cell Signaling Technology (CST)) (Figure 1a), the NANOG signal was detected in the form of one or two punctae located in the cytoplasm near the cell nucleus (Figure 1b and Figure S1). The apparent perinuclear NANOG localization was confirmed using another anti-NANOG commercial antibody (#ab109250, Abcam) (Figure 1a,b and Figure S1).

**Figure 2:**
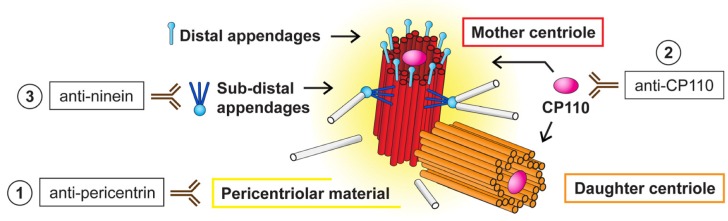
Schematic overview of the different centriolar structures and centrosome-specific antibodies used in this study. The centrosome is a microtubule organizing center (MTOC) composed of two centrioles—older mother centriole (red) and younger daughter centriole (orange). These two centrioles differ structurally [40,41,42]. The mother centriole has distal and subdistal appendages (blue) [43], which promote membrane docking during cilia initiation [44] and microtubule anchoring [45,46], respectively. Both centrioles are surrounded by pericentriolar material (yellow). Binding of the antibodies used in the study to distinguish specific centrosomal parts is depicted: (1) anti-pericentrin antibody is suitable to detect the whole centrosome, (2) anti-CP110 antibody recognizes distal ends of individual centrioles, and (3) anti-ninein is specific for subdistal appendages of the mother centriole.

**Figure 3:**
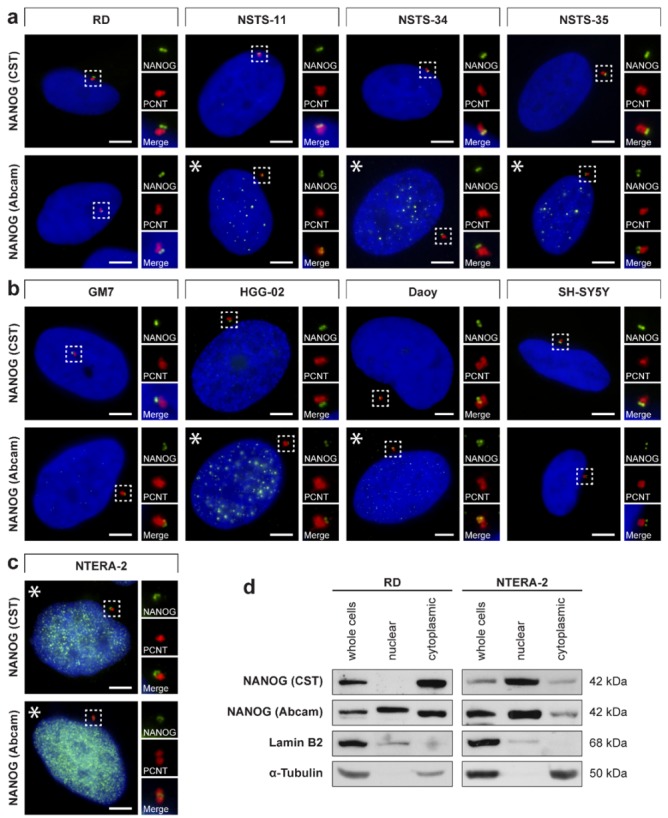
Centrosomal localization of NANOG in tumor cell lines and Western blotting of the nuclear and cytoplasmic fractions. The centrosomal localization of NANOG (green) was detected in (**a**) rhabdomyosarcoma cell lines, (**b**) cell lines of neurogenic origin, and (**c**) NTERA-2 pluripotent embryonal carcinoma cell line using two anti-NANOG antibodies (#4903, Cell Signaling Technology (CST) and #ab109250, Abcam) and anti-pericentrin antibody (PCNT; red). NANOG was also detected in the cell nucleus in the NTERA-2 cell line and some other tumor cell lines (indicated by asterisks). The nuclei were counterstained with Hoechst 33342 (blue). For each image, a region of interest is indicated by the dashed box, and the respective close-ups are provided on the right. Scale bars, 5 μm. (**d**) Western blot analysis revealed the presence of a substantial pool of cytoplasmic NANOG in both RD and NTERA-2 cell lines. Nuclear/cytoplasmic fractionation followed by immunoblotting was performed using two independent anti-NANOG commercial antibodies (#4903, Cell Signaling Technology (CST) and #ab109250, Abcam). Lamin B2 and α-tubulin served as controls of the nuclear fraction and cytoplasmic fraction purity, respectively. Western blots of biological replicates are provided in Figure S5.

Surprisingly, the NANOG cytoplasmic signal resembled centrosomes in their localization and quantity. To investigate whether extranuclear NANOG colocalizes with centrosomes, we performed double indirect immunofluorescence using an anti-pericentrin antibody specific for the detection of centrosomes (Figure 2) combined with each of the two independent anti-NANOG commercial antibodies (Figure 3a–c). In addition to rhabdomyosarcoma cell lines, we also examined established and patient-derived tumor cell lines of neurogenic origin, as well as the NTERA-2 human pluripotent embryonal carcinoma cell line, which served as a positive control of nuclear NANOG expression [39]. Indeed, the localization of the fluorescence signal for NANOG colocalized with centrosomes in each of the nine examined tumor cell lines (Figure 3a–c). While rhabdomyosarcoma cell lines (Figure 3a and Figure S2) and tumor cell lines of neurogenic origin (Figure 3b and Figure S3) showed primarily centrosomal and rarely weak nuclear (indicated by asterisks) localization of NANOG, strong NANOG expression was observed both in the cell nucleus (indicated by asterisks) and at the centrosomes of the control NTERA-2 cell line (Figure 3c and Figure S4).

Validation of the results achieved through indirect immunofluorescence was carried out by immunoblot analysis of the nuclear and cytoplasmic fractions of RD and NTERA-2 cells that differed in the nuclear expression of NANOG (Figure 3d). In agreement with the indirect immunofluorescence results, the presence of NANOG-specific bands was detected not only in the whole-cell lysate and the nuclear fraction but also in the cytoplasmic fraction of both RD and NTERA-2 cell lines. The weaker intensity of the NANOG-specific band detected in the nuclear fraction of RD cells compared with that of NTERA-2 cells is also completely in accordance with our findings obtained by indirect immunofluorescence, as reported above.

**Figure 4:**
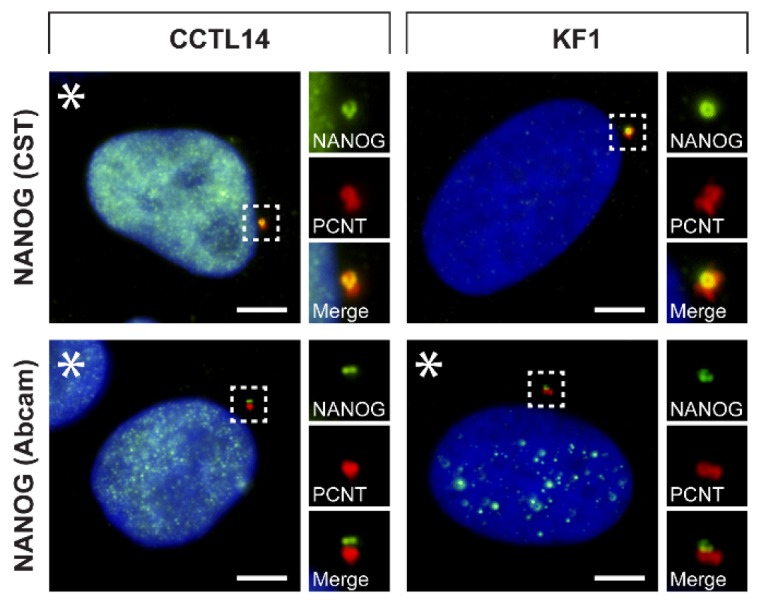
Centrosomal localization of NANOG in hESCs and normal fibroblast cells. The centrosomal localization of NANOG (green) was detected in CCTL14 hESCs and KF1 normal fibroblast cells using two anti-NANOG commercial antibodies (#4903, Cell Signaling Technology (CST) and #ab109250, Abcam) and anti-pericentrin antibody (PCNT; red). NANOG was also detected in the cell nucleus in the CCTL14 cell line and rarely in the KF1 cell line (indicated by asterisks). The nuclei were counterstained with Hoechst 33342 (blue). For each image, a region of interest is indicated by the dashed box, and the respective close-ups are provided on the right. Scale bars, 5 μm.

Our results in nine tumor cell lines indicated that the centrosomal localization of NANOG might be common among tumor cells of various origins. To further investigate whether NANOG may also be present in centrosomes of non-cancerous cell types, we employed double indirect immunofluorescence of pluripotent CCTL14 hESCs and the KF1 normal skin fibroblast cell line using both anti-NANOG commercial antibodies and anti-pericentrin antibody (Figure 4 and Figure S4). Again, the colocalization of NANOG and pericentrin was observed in both cell lines. As expected, NANOG was also detected in the nucleus (indicated by asterisks) in CCTL14 hESCs but rarely in KF1 fibroblasts.

### 3.2. Validation of NANOG Centrosomal Signal Specificity and Anti-NANOG Antibodies

**Figure 5:**
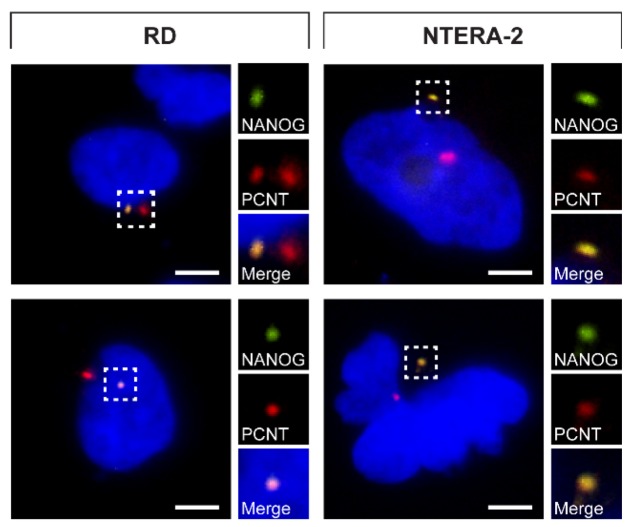
NANOG-GFP colocalization with centrosomes. NANOG-GFP (green) was detected in colocalization with the centrosomes stained by anti-pericentrin antibody (PCNT; red). The nuclei were counterstained with Hoechst 33342 (blue). For each image, a region of interest is indicated by the dashed box, and the respective close-ups are provided on the right. Scale bars, 5 μm.

Given the intriguing centrosomal localization of NANOG protein detected among various cell types, we wanted to rule out the possibility that our observations were caused by a nonspecific binding of both independent anti-NANOG antibodies used for this study. To avoid the need for antibodies for NANOG protein visualization, RD and NTERA-2 cells were transiently transfected with a GFP-tagged NANOG (NANOG-GFP) expression vector. Subsequently, the detection of NANOG-GFP protein fluorescence was used to evaluate the colocalization of NANOG with centrosomes immunostained using anti-pericentrin antibody. As apparent from the immunofluorescence images, the NANOG-GFP signal was undoubtedly localized within the centrosome (Figure 5), which strongly supports the results obtained using anti-NANOG antibodies. However, in cases where two centrosomes were detected due to centrosome duplication, the NANOG-GFP signal was only present at one of the centrosomes in the cell.

In addition, the specificity of the anti-NANOG antibodies was further examined by two complementary approaches. First, the immunogen sequence of the #ab109250 antibody was compared with the sequences of centrosomal proteins using the Blast^®^ (https://blast.ncbi.nlm.nih.gov/Blast.cgi) and UniProt (https://www.UniProt.org/align/) protein databases. Although the immunogen sequence of both antibodies is proprietary, we received information about the #ab109250 antibody on request from the Abcam manufacturer. Nevertheless, no identity with centrosomal proteins sequences was found. To validate the binding specificity of the anti-NANOG antibody, NTERA-2 cell lysate was immunoprecipitated using #ab109250 antibody, and the bound proteins were analyzed by mass spectrometry. The NANOGP8 (Q6NSW7) peptide was identified as the most prominent, although a sequence identical for all NANOGP8, NANOG (Q9H9S0), and NANOGP1 (Q8N7R0) was also found (Table S1). These results confirmed the binding specificity of the anti-NANOG #ab109250 antibody and pointed to the expression of NANOGP8 protein along with NANOG and/or NANOGP1 in NTERA-2 cells. Notably, #4903 antibody is not suitable for immunoprecipitation; therefore, this antibody was not tested by mass spectrometry.

### 3.3. Analysis of NANOG and NANOGP8 Gene Expression

**Figure 6:**
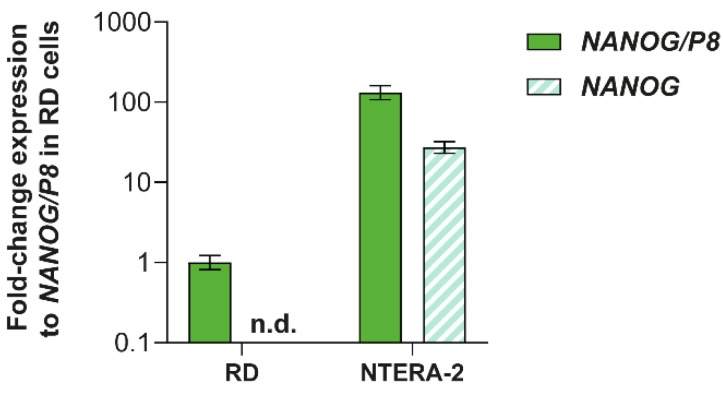
Expression levels of *NANOG* and *NANOGP8* in RD and NTERA-2 cell lines. The relative expression was analyzed by RT-qPCR using *NANOG*-specific primers (*NANOG*) and primers, which detect both *NANOGP8* and *NANOG* transcripts (*NANOG/P8*). Expression was normalized to the endogenous control, *HSP90AB1*, and the fold-change expression values are presented as the mean ± SD from three biological replicates.

Based on the mass spectrometry results, we next investigated the relative expression levels of *NANOG* and *NANOGP8* genes using RT-qPCR. While *NANOG*-specific primers revealed *NANOG* gene expression only in the NTERA-2 cell line, data obtained using *NANOGP8* primers, which may also amplify *NANOG* gene transcripts, indicated that *NANOGP8* is expressed in both NTERA-2 and RD cell lines (Figure 6). The results further showed that *NANOGP8* is abundantly expressed in RD cells. Together with mass spectrometry, these data suggest that the protein might in fact be the NANOGP8 protein, which is localized at the centrosomes.

### 3.4. NANOG Colocalizes with the Mother Centriole

**Figure 7:**
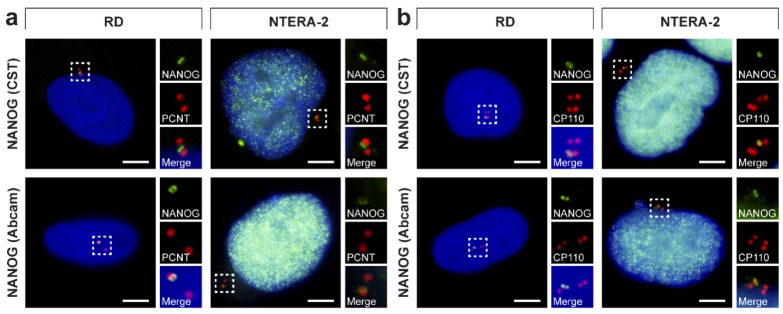
Interaction of NANOG with only one centriole within a cell. NANOG (green; #4903, Cell Signaling Technology (CST) and #ab109250, Abcam) was detected in only (**a**) one centrosome (pericentrin, PCNT; red) and (**b**) one centriole (CP110; red) during the interphase of RD and NTERA-2 cells. The nuclei were counterstained with Hoechst 33342 (blue). For each image, a region of interest is indicated by the dashed box, and the respective close-ups are provided on the right. Scale bars, 5 μm.

As briefly noted above (Figure 5), during the immunofluorescence analysis, we noted an apparent association of the NANOG signal with only one centrosome from the centrosome pair present in interphase cells (Figure 7a). These results led us to perform a detailed analysis of the interaction of NANOG with centrosomal components. First, we employed double indirect immunofluorescence of anti-CP110 specific for the detection of distal end of the centriole (Figure 2) together with each of the anti-NANOG antibodies used in this study (Figure 7b). Regardless of the anti-NANOG antibody used, in both examined RD and NTERA-2 cell lines, the extranuclear signal of NANOG was localized to only one of the centrioles within individual interphase cells. Therefore, the next step of this study was to determine which centriole the NANOG protein associates with.

**Figure 8:**
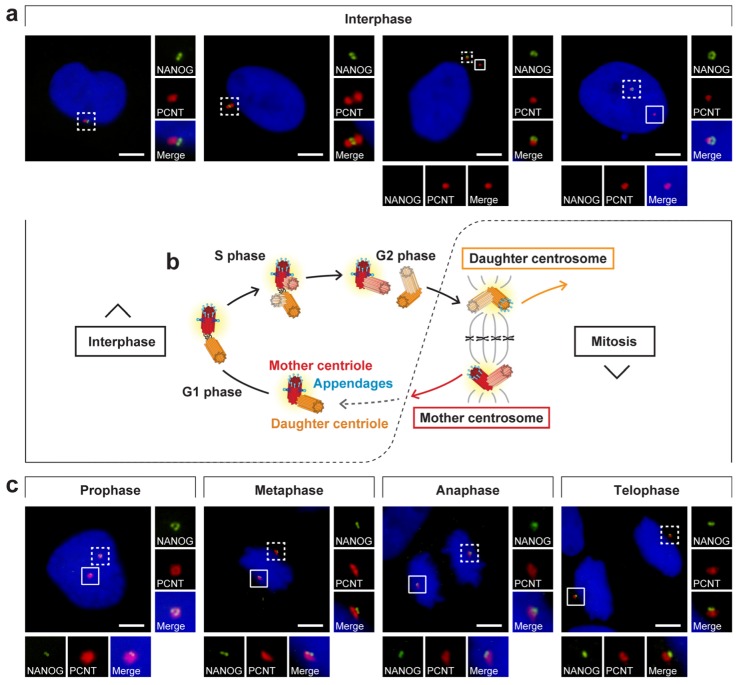
NANOG localization during the cell cycle. (**a**,**c**) Analysis of NANOG during the cell cycle by double indirect immunofluorescence. NANOG (green; #4903, Cell Signaling Technology (CST)) colocalized with one centrosome (pericentrin, PCNT; red) only during interphase (**a**) but with both centrosomes (pericentrin, PCNT; red) during mitosis (**c**). The nuclei were counterstained with Hoechst 33342 (blue). For each image, regions of interest are indicated by the dashed and solid boxes, and the close-ups are provided on the right and below, respectively. Scale bars, 5 μm. (**b**) Schematic visualization of the centrosome duplication cycle: The centrosome is copied only once per cell cycle following cytokinesis. The centrosome duplication cycle results in two daughter cells with different ages of centrosomes: one that keeps the mother centrosome with the old mother centriole and the other cell that inherits the daughter centrosome, which comprises the young mother centriole [51,52]. Distal appendages (light blue) remain on the mother centriole during the whole cell cycle and appear during mitosis or shortly after mitosis on the older centriole of the daughter centrosome [43,53]. After mitosis, the newly formed mother centriole assembles subdistal appendages (blue) [43,53].

Centrosome duplication is coordinated with cell cycle progression [47,48,49,50] and results in two daughter cells: one with the mother centrosome and one with the daughter centrosome (Figure 8b) [51]. Thus, we focused on the presence or absence of the NANOG signal during the cell cycle (Figure 8a,c) to determine whether the centrosomal localization of NANOG is also coordinated with cell cycle progression. In interphase cells, NANOG was detected in only one centrosome (Figure 8a). However, the fluorescence signal of NANOG on the second centrosome was established in mitosis (Figure 8c and Figure S6). Considering the centrosome duplication cycle (Figure 8b) and the differences in the structure of mother and daughter centrioles (Figure 2), these results suggested that appearance of NANOG labelling at the second mitotic spindle pole correlated with centrosome maturation. This led us to conclusion that during interphase, NANOG localized in the centrosome which contains the mother centriole.

**Figure 9:**
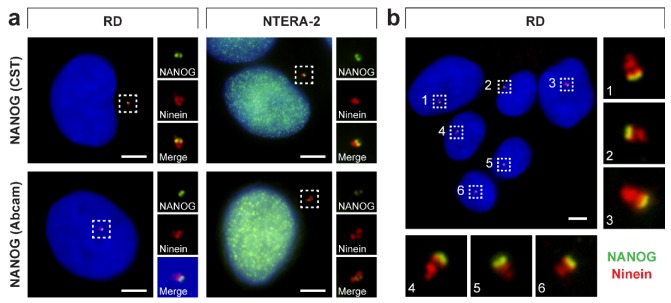
NANOG colocalization with the mother centriole. (**a**) A colocalization of NANOG (green; #4903, Cell Signaling Technology (CST) and #ab109250, Abcam) with the mother centriole was detected using anti-ninein antibody (red), recognizing a protein of the mother centriole subdistal appendages. The nuclei were counterstained with Hoechst 33342 (blue). For each image, regions of interest and the respective close-ups are indicated by the dashed boxes. (**b**) NANOG signal (green; #4903, Cell Signaling Technology) partially colocalized with the “double-spot” signal of ninein (red). The nuclei were counterstained with Hoechst 33342 (blue). Respective close-ups of NANOG and ninein colocalization are indicated by numbers. Scale bars, 5 μm.

To examine whether NANOG interacts with the mother centriole, we performed double indirect immunofluorescence of both anti-NANOG commercial antibodies together with the detection of ninein (Figure 9), which is a mother centriole–specific protein predominantly localized at subdistal appendages of the mother centriole (Figure 2) [54,55,56,57]. These experiments revealed a close spatial proximity of fluorescence signals for NANOG and ninein (Figure 9), which clearly confirms the interaction of NANOG with the mother centriole. Several studies have shown that when a centrosome is imaged from the side view, the immunofluorescence signals of ninein at the mother centriole mostly appear as three foci: an individual spot, which marks the proximal part of the mother centriole, and two closer spots (sometimes merged in a larger signal), which are located at the subdistal appendages of the mother centriole [58,59,60]. Given the observed spatial distribution of NANOG relative to signals of ninein (Figure 9b) and CP110 (Figure 7b), which caps the distal ends of centrioles [30,61], we conclude that NANOG localizes at the distal region of the mother centriole.

## 4. Discussion

Taken together, our results clearly demonstrate the presence of NANOG protein at the centrosomes and indicate that its spatiotemporal localization associates with the mother centriole. This interesting phenomenon was proven in tumor cell lines, as well as non-cancerous hESCs and normal fibroblast cells. Considering the mass spectrometry and RT-qPCR results, this protein was most likely the NANOG paralog NANOGP8, which was localized in centrosomes and might be the cause of the NANOG signal detected within these organelles. Whether this phenomenon is common remains speculative, and further investigation will be needed to distinguish the subcellular localization of NANOG and NANOGP8 proteins, which have nearly 100% homology and have been demonstrated to possess similar reprogramming activity [17].

To date, the atypical centrosomal localization of NANOG was only briefly noted in a study focused on induced pluripotent stem cells [62]. By using the same #4903 Cell Signaling Technology anti-NANOG antibody, the authors observed a perinuclear signal of NANOG in HEK293 cells and amniotic cell-derived iPSCs. However, the study lacks validation of the suggested centrosomal localization of NANOG by any centrosome-specific antibody, and the anti-NANOG antibody specificity has not been examined by proper controls and additional experiments [62]. In this report, we present the first systematic analysis of NANOG colocalization with centrosomes using two independent anti-NANOG commercial antibodies together with antibodies recognizing different centrosomal proteins.

Indeed, our immunofluorescence results undeniably demonstrate the colocalization of NANOG with centrosomes, as visualized by anti-pericentrin, anti-CP110, and anti-ninein antibodies. Importantly, the authenticity of the NANOG signal in centrosomes was independently proven by NANOG-GFP transfection experiments. Surprisingly, cell fractionation further revealed that RD cells, which exhibit only weak nuclear staining of NANOG compared with NTERA-2 cells, contained a substantial amount of NANOG protein present in the cytoplasmic fraction. Given the specific cytoplasmic distribution of NANOG in RD cells, the amount of protein detected in the cytoplasmic fraction generally reflected the centrosome-localized pool of NANOG. Along with the finding that the centrosomal localization of NANOG was observed among various tumor and non-tumor cell types, these results provide the first evidence suggesting a common centrosome-specific role of NANOG. We hypothesize that this function might be maintained even in cells with low/basal levels of NANOG protein. 

Several transcription factors have been previously described to be present at the centrosome [63,64], including recently reported ATF5 [64]. Independent of its role as a transcription factor, ATF5 was demonstrated to be indispensable for proper centrosome assembly and maintenance [64]. To exert its function in the process of pericentriolar material accumulation, ATF5 localizes at the mother centriole in a cell cycle- and centriole age-dependent manner [64]. Interestingly, the reported spatial and temporal profile of ATF5 [64] is in keeping with our observations with NANOG.

**Figure 10:**
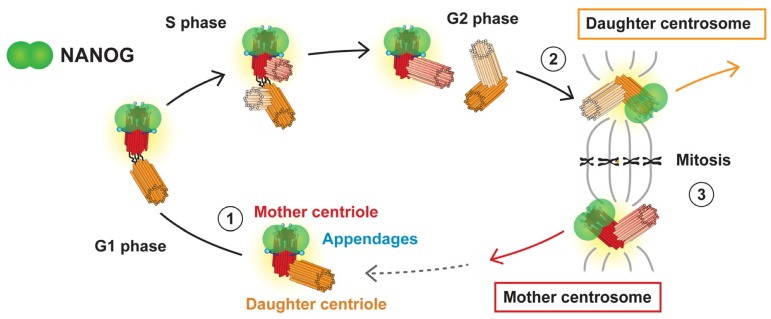
Schematic summarizing the observed spatiotemporal localization of NANOG/NANOGP8 at centrosome during the cell cycle. NANOG (green) is present at the same time as mother centriole appendages: (1) During interphase, NANOG protein colocalizes with the distal region of the mother centriole (red), which carries distal and subdistal appendages (blue). (2) NANOG signal as well as the distal appendages also later appear on the initially daughter centriole (orange) during the G2/mitosis transition. (3) The centrosome duplication cycle results in two daughter cells with different ages of centrosomes, mother and daughter centrosomes, both having the older, mother centriole marked with NANOG protein.

In our study, NANOG was identified at only one centrosome in interphase cells, even after centrosome separation before mitosis. In contrast, the fluorescence signal of NANOG was also detected at the other centrosome of the centrosome pair during mitosis. The presence/absence of NANOG signal at the centrosome during the cell cycle corresponded with the centriole maturation, which is accompanied by the formation of centriole appendages. The association of NANOG protein with the mother centriole was experimentally confirmed by immunofluorescence staining using anti-ninein antibody, which is specific for the mother centriole subdistal appendages. Taken together, these results suggest an intricate centriole maturation-dependent interaction of NANOG with the mother centriole. The main observations of our study regarding NANOG occurrence at the centrosome during the cell cycle are schematically summarized in Figure 10.

A question remains regarding the exact role of NANOG and/or NANOGP8 in centrosomes and why its presence appears to be dependent on the age of centrosomes. Centrosome duplication is an elaborate process in which many proteins are involved [48,49,50]. Duplication of centrosomes begins by separation of mother and daughter centrioles and formation of a procentriole adjacent to both centrioles during the G1 phase [65,66]. Procentrioles elongate through S and G2 phases followed by maturation of centrioles. The final step of centriole maturation is the building of distal and subdistal appendages on their distal ends. The presence of distal appendages depends on ODF1 [50,67] and C2cd3 [68] followed by Cep83, which then recruits other proteins, including Cep89, SCLT1, FBF1, and Cep164 [44,50]. The formation of subdistal appendages is initiated by two groups of proteins. The first group is led by ODF2 [50,58,69,70] followed by the recruitment of various proteins, including CCDC120, CCDC68, trichoplein [50,59], CEP128, and centriolin [69]. The second group is led by ninein, followed by Kif2a, p150^Glued^ and CEP170 [59,69,70], centriolin, and ε-tubulin [71].

The formation of distal and subdistal appendages is a highly complicated and still poorly understood process. Nevertheless, the newly formed mother centriole lacks distal and subdistal appendages until the G2/M phase transition and G1 phase of the next cell cycle, respectively [43,53]. Corroborating the functional differences between the mother and daughter centrosomes, a strong correlation between centrosome age and cell fate has been demonstrated during the cell division of stem cells [72,73]. In addition, proper orientation of centrosomes and thus mitotic spindle orientation also appear to be strictly orchestrated for the maintenance of stemness [72,74]. Our results describing the presence/absence of the NANOG signal during the cell cycle (Figure 8a,c) correspond with the occurrence of centriole appendages. Therefore, all these results indicate the association of NANOG protein or its NANOGP8 paralog with the process of centriole maturation. Such connections have never been described or functionally explored. The hypothesis that NANOG, a protein crucially involved in the regulation of pluripotency and stemness, might be directly involved in the regulation of centrosome and/or mitotic spindle assembly provokes many scientific questions that should be pursued in future studies. For this reason, our further research will investigate the possible function of NANOG/NANOGP8 associated with centrosomes.

## Figures and Tables

**Table 1:** Description of cell lines.

Cell Line	Tissue Type	Source
Tumor Cell Lines		
RD	Embryonal rhabdomyosarcoma	ECACC, Cat. No. 85111502
NSTS-11	Rhabdomyosarcoma	[25]
NSTS-34	Rhabdomyosarcoma	Established in the laboratory
NSTS-35	Rhabdomyosarcoma	Established in the laboratory
GM7	Glioblastoma multiforme	[26]
HGG-02	Glioblastoma multiforme	[27]
Daoy	Medulloblastoma	ATCC, HTB-186^TM^
SH-SY5Y	Neuroblastoma	ECACC, Cat. No. 94030304
NTERA-2	Embryonal carcinoma	ECACC, Cat. No. 01071221
**Non-Tumor Cell Lines**		
CCTL14	Human embryonal stem cells	[24]
KF1	Normal fibroblasts	[28]

**Table 2:** Antibodies used in the study.

Primary Antibodies					
Antigen	Isotype	Clone (Cat. No.)	Manufacturer	Dilution	
				IF	WB
Nanog	Rb IgG	D73G4 (4903)	CST	1:200	1:2000
Nanog	Rb IgG	EPR2027(2) (ab109250)	Abcam	1:200	1:2000
Pericentrin	Rb IgG	- (ab4448)	Abcam	1:400	-
CP110	Mo	-	-	1:5	-
Ninein	Mo IgG2a	79.160-7 (41-3400)	Thermo Fisher	1:800	-
α-Tubulin	Mo IgG1	DM1A (ab7291)	Abcam	-	1:10,000
Lamin B2	Rb IgG	D8P3U (12255)	CST	-	1:400
**Secondary Antibodies**					
**Host**	**Specificity**	**Conjugate**	**Manufacturer**	**Dilution**	
				**IF**	**WB**
Donkey	anti-Mo IgG	Alexa Fluor 488	Life Technologies	1:200	-
Donkey	anti-Rb IgG	Alexa Fluor 488	Life Technologies	1:200	-
Donkey	anti-Mo IgG	Alexa Fluor 568	Life Technologies	1:200	-
Donkey	anti-Rb IgG	Alexa Fluor 568	Life Technologies	1:200	-
Horse	anti-Mo IgG	HRP	CST	-	1:5000
Goat	anti-Rb IgG	HRP	CST	-	1:5000

*Mo* mouse, *Rb* rabbit, *HRP* horseradish peroxidase, *IF* immunofluorescence, *WB* Western blotting, *CST* Cell Signaling Technology.

**Table 3:** Primers used in the study.

Gene	Gene Symbol	Primer Sequence
Nanog homeobox	*NANOG*	F: 5′-TTCATTATAAATCTAGAGACTCCAGGA-3′
		R: 5′-CTTTGGGACTGGTGGAAGAATC-3′
Nanog homeobox/Nanog homeobox retrogene P8 *	*NANOG/P8*	F: 5′-GCAGAGAAGAGTGTCG-3′
		R: 5′-AGCTGGGTGGAAGAGAACACAG-3′
Heat shock protein 90 alpha family class B member 1	*HSP90AB1*	F: 5′-CGCATGAAGGAGACACAGAA-3′
		R: 5′-TCCCATCAAATTCCTTGAGC-3′

*F* forward primer, *R* reverse primer; * Indicates that this set of primers matches to both NANOG and NANOGP8 sequences.

## Supplementary Materials

The following are available online: https://www.mdpi.com/2073-4409/9/3/692/s1. Figure S1: Unmerged images from Figure 1b; Figure S2: Unmerged images from Figure 3a; Figure S3: Unmerged images from Figure 3b; Figure S4: Unmerged images from Figure 3 and Figure 4; Figure S5: Western blot images of NANOG immunodetection in three biological replicates of the nuclear and cytoplasmic fractions of RD and NTERA-2 cells; Figure S6: NANOG localization during the cell cycle; Table S1. Validation of anti-NANOG #ab109250 antibody specificity by mass spectrometry of immunoprecipitated NTERA-2 cells.

## References

[1] Chambers I., Colby D., Robertson M., Nichols J., Lee S., Tweedie S., Smith A. (2003). Functional Expression Cloning of Nanog, a Pluripotency Sustaining Factor in Embryonic Stem Cells. Cell.

[2] Mitsui K., Tokuzawa Y., Itoh H., Segawa K., Murakami M., Takahashi K., Maruyama M., Maeda M., Yamanaka S. (2003). The Homeoprotein Nanog Is Required for Maintenance of Pluripotency in Mouse Epiblast and ES Cells. Cell.

[3] Silva J., Nichols J., Theunissen T.W., Guo G., van Oosten A.L., Barrandon O., Wray J., Yamanaka S., Chambers I., Smith A. (2009). Nanog is the gateway to the pluripotent ground state. Cell.

[4] Hart A.H., Hartley L., Parker K., Ibrahim M., Looijenga L.H.J., Pauchnik M., Chow C.W., Robb L. (2005). The pluripotency homeobox gene NANOG is expressed in human germ cell tumors. Cancer.

[5] Zbinden M., Duquet A., Lorente-Trigos A., Ngwabyt S.-N., Borges I., Ruiz i Altaba A. (2010). NANOG regulates glioma stem cells and is essential in vivo acting in a cross-functional network with GLI1 and p53. EMBO J..

[6] Nagata T., Shimada Y., Sekine S., Hori R., Matsui K., Okumura T., Sawada S., Fukuoka J., Tsukada K. (2014). Prognostic significance of NANOG and KLF4 for breast cancer. Breast Cancer.

[7] Santaliz-Ruiz L.E., Xie X., Old M., Teknos T.N., Pan Q. (2014). Emerging Role of Nanog in Tumorigenesis and Cancer Stem Cells. Int. J. Cancer J. Int. Cancer.

[8] Fan Z., Li M., Chen X., Wang J., Liang X., Wang H., Wang Z., Cheng B., Xia J. (2017). Prognostic Value of Cancer Stem Cell Markers in Head and Neck Squamous Cell Carcinoma: A Meta-analysis. Sci. Rep..

[9] Freitag D., McLean A.L., Simon M., Koch A., Grube S., Walter J., Kalff R., Ewald C. (2017). NANOG overexpression and its correlation with stem cell and differentiation markers in meningiomas of different WHO grades. Mol. Carcinog..

[10] Rasti A., Mehrazma M., Madjd Z., Abolhasani M., Zanjani L.S., Asgari M. (2018). Co-expression of Cancer Stem Cell Markers OCT4 and NANOG Predicts Poor Prognosis in Renal Cell Carcinomas. Sci. Rep..

[11] Nichols J., Zevnik B., Anastassiadis K., Niwa H., Klewe-Nebenius D., Chambers I., Schöler H., Smith A. (1998). Formation of pluripotent stem cells in the mammalian embryo depends on the POU transcription factor Oct4. Cell.

[12] Avilion A.A., Nicolis S.K., Pevny L.H., Perez L., Vivian N., Lovell-Badge R. (2003). Multipotent cell lineages in early mouse development depend on SOX2 function. Genes Dev..

[13] Pan G., Thomson J.A. (2007). Nanog and transcriptional networks in embryonic stem cell pluripotency. Cell Res..

[14] Hyslop L., Stojkovic M., Armstrong L., Walter T., Stojkovic P., Przyborski S., Herbert M., Murdoch A., Strachan T., Lako M. (2005). Downregulation of NANOG induces differentiation of human embryonic stem cells to extraembryonic lineages. Stem Cells Dayt. Ohio.

[15] Schorle H., Nettersheim D. (2012). NANOG (Nanog homeobox). Atlas Genet. Cytogenet. Oncol. Haematol..

[16] Booth H.A.F., Holland P.W.H. (2004). Eleven daughters of NANOG. Genomics.

[17] Palla A.R., Piazzolla D., Abad M., Li H., Dominguez O., Schonthaler H.B., Wagner E.F., Serrano M. (2014). Reprogramming activity of NANOGP8, a NANOG family member widely expressed in cancer. Oncogene.

[18] Zhang J., Wang X., Li M., Han J., Chen B., Wang B., Dai J. (2006). NANOGP8 is a retrogene expressed in cancers. FEBS J..

[19] Ambady S., Malcuit C., Kashpur O., Kole D., Holmes W.F., Hedblom E., Page R.L., Dominko T. (2010). Expression of NANOG and NANOGP8 in a variety of undifferentiated and differentiated human cells. Int. J. Dev. Biol..

[20] Do H.-J., Lim H.-Y., Kim J.-H., Song H., Chung H.-M., Kim J.-H. (2007). An intact homeobox domain is required for complete nuclear localization of human Nanog. Biochem. Biophys. Res. Commun..

[21] Chang D.F., Tsai S.C., Wang X.C., Xia P., Senadheera D., Lutzko C. (2009). Molecular Characterization of the Human NANOG Protein. Stem Cells.

[22] Gu T.-T., Liu S.-Y., Zheng P.-S. (2012). Cytoplasmic NANOG-Positive Stromal Cells Promote Human Cervical Cancer Progression. Am. J. Pathol..

[23] van Schaijik B., Davis P.F., Wickremesekera A.C., Tan S.T., Itinteang T. (2018). Subcellular localisation of the stem cell markers OCT4, SOX2, NANOG, KLF4 and c-MYC in cancer: A review. J. Clin. Pathol..

[24] Dvorak P., Dvorakova D., Koskova S., Vodinska M., Najvirtova M., Krekac D., Hampl A. (2005). Expression and Potential Role of Fibroblast Growth Factor 2 and Its Receptors in Human Embryonic Stem Cells. STEM CELLS.

[25] Sana J., Zambo I., Skoda J., Neradil J., Chlapek P., Hermanova M., Mudry P., Vasikova A., Zitterbart K., Hampl A. (2011). CD133 Expression and Identification of CD133/nestin Positive Cells in Rhabdomyosarcomas and Rhabdomyosarcoma Cell Lines. Anal. Cell. Pathol. Amst..

[26] Loja T., Chlapek P., Kuglik P., Pesakova M., Oltova A., Cejpek P., Veselska R. (2009). Characterization of a GM7 glioblastoma cell line showing CD133 positivity and both cytoplasmic and nuclear localization of nestin. Oncol. Rep..

[27] Veselska R., Skoda J., Loja T., Zitterbart K., Pavelka Z., Smardova J., Valaskova I., Hermanova M., Sterba J. (2010). An unusual loss of EGFR gene copy in glioblastoma multiforme in a child: A case report and analysis of a successfully derived HGG-02 cell line. Childs Nerv. Syst. ChNS Off. J. Int. Soc. Pediatr. Neurosurg..

[28] Veselska R., Janisch R. Reaction of the Skin Fibroblast Cytoskeleton to Micromanipulation Interventions–ScienceDirect. https://www.sciencedirect.com/science/article/pii/S1047847701944326.

[29] Mikulenkova E., Neradil J., Zitterbart K., Sterba J., Veselska R. (2015). Overexpression of the ∆Np73 isoform is associated with centrosome amplification in brain tumor cell lines. Tumour Biol. J. Int. Soc. Oncodevelopmental Biol. Med..

[30] Kleylein-Sohn J., Westendorf J., Le Clech M., Habedanck R., Stierhof Y.-D., Nigg E.A. (2007). Plk4-induced centriole biogenesis in human cells. Dev. Cell.

[31] Wiśniewski J.R., Zougman A., Nagaraj N., Mann M. (2009). Universal sample preparation method for proteome analysis. Nat. Methods.

[32] Holman J.D., Tabb D.L., Mallick P. (2014). Employing ProteoWizard to Convert Raw Mass Spectrometry Data. Curr. Protoc. Bioinforma..

[33] McIlwain S., Tamura K., Kertesz-Farkas A., Grant C.E., Diament B., Frewen B., Howbert J.J., Hoopmann M.R., Käll L., Eng J.K. (2014). Crux: Rapid open source protein tandem mass spectrometry analysis. J. Proteome Res..

[34] The M., MacCoss M.J., Noble W.S., Käll L. (2016). Fast and Accurate Protein False Discovery Rates on Large-Scale Proteomics Data Sets with Percolator 3.0. J. Am. Soc. Mass Spectrom..

[35] Millikin R.J., Solntsev S.K., Shortreed M.R., Smith L.M. (2018). Ultrafast Peptide Label-Free Quantification with FlashLFQ. J. Proteome Res..

[36] Silva J.C., Gorenstein M.V., Li G.-Z., Vissers J.P.C., Geromanos S.J. (2006). Absolute quantification of proteins by LCMSE: A virtue of parallel MS acquisition. Mol. Cell. Proteomics MCP.

[37] Livak K.J., Schmittgen T.D. (2001). Analysis of relative gene expression data using real-time quantitative PCR and the 2(-Delta Delta C(T)) Method. Methods San Diego Calif.

[38] Ma X., Wang B., Wang X., Luo Y., Fan W. (2018). NANOGP8 is the key regulator of stemness, EMT, Wnt pathway, chemoresistance, and other malignant phenotypes in gastric cancer cells. PLoS ONE.

[39] Hu Q., Khanna P., Ee Wong B.S., Lin Heng Z.S., Subhramanyam C.S., Thanga L.Z., Sing Tan S.W., Baeg G.H. (2018). Oxidative stress promotes exit from the stem cell state and spontaneous neuronal differentiation. Oncotarget.

[40] Paintrand M., Moudjou M., Delacroix H., Bornens M. (1992). Centrosome organization and centriole architecture: Their sensitivity to divalent cations. J. Struct. Biol..

[41] Sonnen K.F., Schermelleh L., Leonhardt H., Nigg E.A. (2012). 3D-structured illumination microscopy provides novel insight into architecture of human centrosomes. Biol. Open.

[42] Nigg E.A., Holland A.J. (2018). Once and only once: Mechanisms of centriole duplication and their deregulation in disease. Nat. Rev. Mol. Cell Biol..

[43] Uzbekov R., Alieva I. (2018). Who are you, subdistal appendages of centriole?. Open Biol..

[44] Tanos B.E., Yang H.-J., Soni R., Wang W.-J., Macaluso F.P., Asara J.M., Tsou M.-F.B. (2013). Centriole distal appendages promote membrane docking, leading to cilia initiation. Genes Dev..

[45] Vorobjev I.A., Nadezhdina E.S. (1987). The centrosome and its role in the organization of microtubules. Int. Rev. Cytol..

[46] Bornens M. (2002). Centrosome composition and microtubule anchoring mechanisms. Curr. Opin. Cell Biol..

[47] Hinchcliffe E.H. (2001). “It Takes Two to Tango”: Understanding how centrosome duplication is regulated throughout the cell cycle. Genes Dev..

[48] Loncarek J., Khodjakov A. (2009). Ab ovo or de novo? Mechanisms of centriole duplication. Mol. Cells.

[49] Fujita H., Yoshino Y., Chiba N. (2015). Regulation of the centrosome cycle. Mol. Cell. Oncol..

[50] Loncarek J., Bettencourt-Dias M. (2018). Building the right centriole for each cell type. J. Cell Biol..

[51] Stearns T. (2001). Centrosome duplication. A centriolar pas de deux. Cell.

[52] Reina J., Gonzalez C. (2014). When fate follows age: Unequal centrosomes in asymmetric cell division. Philos. Trans. R. Soc. Lond. B. Biol. Sci..

[53] Vorobjev I.A., Chentsov Y.S. (1982). Centrioles in the cell cycle. I. Epithelial cells. J. Cell Biol..

[54] Piel M., Meyer P., Khodjakov A., Rieder C.L., Bornens M. (2000). The respective contributions of the mother and daughter centrioles to centrosome activity and behavior in vertebrate cells. J. Cell Biol..

[55] Mogensen M.M., Malik A., Piel M., Bouckson-Castaing V., Bornens M. (2000). Microtubule minus-end anchorage at centrosomal and non-centrosomal sites: The role of ninein. J. Cell Sci..

[56] Ou Y.Y., Mack G.J., Zhang M., Rattner J.B. (2002). CEP110 and ninein are located in a specific domain of the centrosome associated with centrosome maturation. J. Cell Sci..

[57] Lee M., Rhee K. (2015). Determination of Mother Centriole Maturation in CPAP-Depleted Cells Using the Ninein Antibody. Endocrinol. Metab. Seoul Korea.

[58] Tateishi K., Yamazaki Y., Nishida T., Watanabe S., Kunimoto K., Ishikawa H., Tsukita S. (2013). Two appendages homologous between basal bodies and centrioles are formed using distinct Odf2 domains. J. Cell Biol..

[59] Huang N., Xia Y., Zhang D., Wang S., Bao Y., He R., Teng J., Chen J. (2017). Hierarchical assembly of centriole subdistal appendages via centrosome binding proteins CCDC120 and CCDC68. Nat. Commun..

[60] Kashihara H., Chiba S., Kanno S., Suzuki K., Yano T., Tsukita S. (2019). Cep128 associates with Odf2 to form the subdistal appendage of the centriole. Genes Cells.

[61] Fu J., Glover D.M. (2012). Structured illumination of the interface between centriole and peri-centriolar material. Open Biol..

[62] Wilson P.G., Payne T. (2014). Genetic reprogramming of human amniotic cells with episomal vectors: Neural rosettes as sentinels in candidate selection for validation assays. PeerJ.

[63] Chuang L.S.H., Lai S.K., Murata-Hori M., Yamada A., Li H.-Y., Gunaratne J., Ito Y. (2012). RUNX3 interactome reveals novel centrosomal targeting of RUNX family of transcription factors. Cell Cycle Georget. Tex.

[64] Madarampalli B., Yuan Y., Liu D., Lengel K., Xu Y., Li G., Yang J., Liu X., Lu Z., Liu D.X. (2015). ATF5 Connects the Pericentriolar Materials to the Proximal End of the Mother Centriole. Cell.

[65] Uzbekov R.E. (2007). Centriole duplication in PE (SPEV) cells starts before the beginning of the DNA replication. Biochem. Mosc. Suppl. Ser. Membr. Cell Biol..

[66] Gupta A., Tsuchiya Y., Ohta M., Shiratsuchi G., Kitagawa D. (2017). NEK7 is required for G1 progression and procentriole formation. Mol. Biol. Cell.

[67] Singla V., Romaguera-Ros M., Garcia-Verdugo J.M., Reiter J.F. (2010). Ofd1, a human disease gene, regulates the length and distal structure of centrioles. Dev. Cell.

[68] Ye X., Zeng H., Ning G., Reiter J.F., Liu A. (2014). C2cd3 is critical for centriolar distal appendage assembly and ciliary vesicle docking in mammals. Proc. Natl. Acad. Sci. USA.

[69] Galati D.F., Mitchell B.J., Pearson C.G. (2016). Subdistal Appendages Stabilize the Ups and Downs of Ciliary Life. Dev. Cell.

[70] Mazo G., Soplop N., Wang W.-J., Uryu K., Tsou M.-F.B. (2016). Spatial Control of Primary Ciliogenesis by Subdistal Appendages Alters Sensation-Associated Properties of Cilia. Dev. Cell.

[71] Chang P., Giddings T.H., Winey M., Stearns T. (2003). Epsilon-tubulin is required for centriole duplication and microtubule organization. Nat. Cell Biol..

[72] Yamashita Y.M., Mahowald A.P., Perlin J.R., Fuller M.T. (2007). Asymmetric Inheritance of Mother Versus Daughter Centrosome in Stem Cell Division. Science.

[73] Pelletier L., Yamashita Y.M. (2012). Centrosome asymmetry and inheritance during animal development. Curr. Opin. Cell Biol..

[74] Yamashita Y.M., Jones D.L., Fuller M.T. (2003). Orientation of asymmetric stem cell division by the APC tumor suppressor and centrosome. Science.

