# Supplementary material for: NANOG/NANOGP8 Localizes at the Centrosome and is Spatiotemporally Associated with Centriole Maturation
Source: Cells. 2020 Mar 11;9(3):692. doi: 10.3390/cells9030692 (PMC7140602; doi:10.3390/cells9030692)
Supplement: Supplementary file 1 [file cells-09-00692-s001.pdf]

Supplementary Materials

NANOG/NANOGP8 Localizes at the Centrosome and Is Spatiotemporally Associated with Centriole Maturation

Erika Mikulenkova, Jakub Neradil, Ondrej Vymazal, Jan Skoda and Renata Veselska

Contents

Supplementary Figures

Figure S1. Unmerged images from Figure 1b.

Figure S2. Unmerged images from Figure 3a.

Figure S3. Unmerged images from Figure 3b.

Figure S4. Unmerged images from Figure 3c and Figure 4.

Figure S5. Western blot images of NANOG immunodetection in three biological replicates of the nuclear and cytoplasmic fractions of RD and NTERA-2 cells.

Figure S6. NANOG localization during the cell cycle.

Supplementary Tables

Table S1. Validation of anti-NANOG #ab109250 antibody specificity by mass spectrometry of immunoprecipitated NTERA-2 cells.

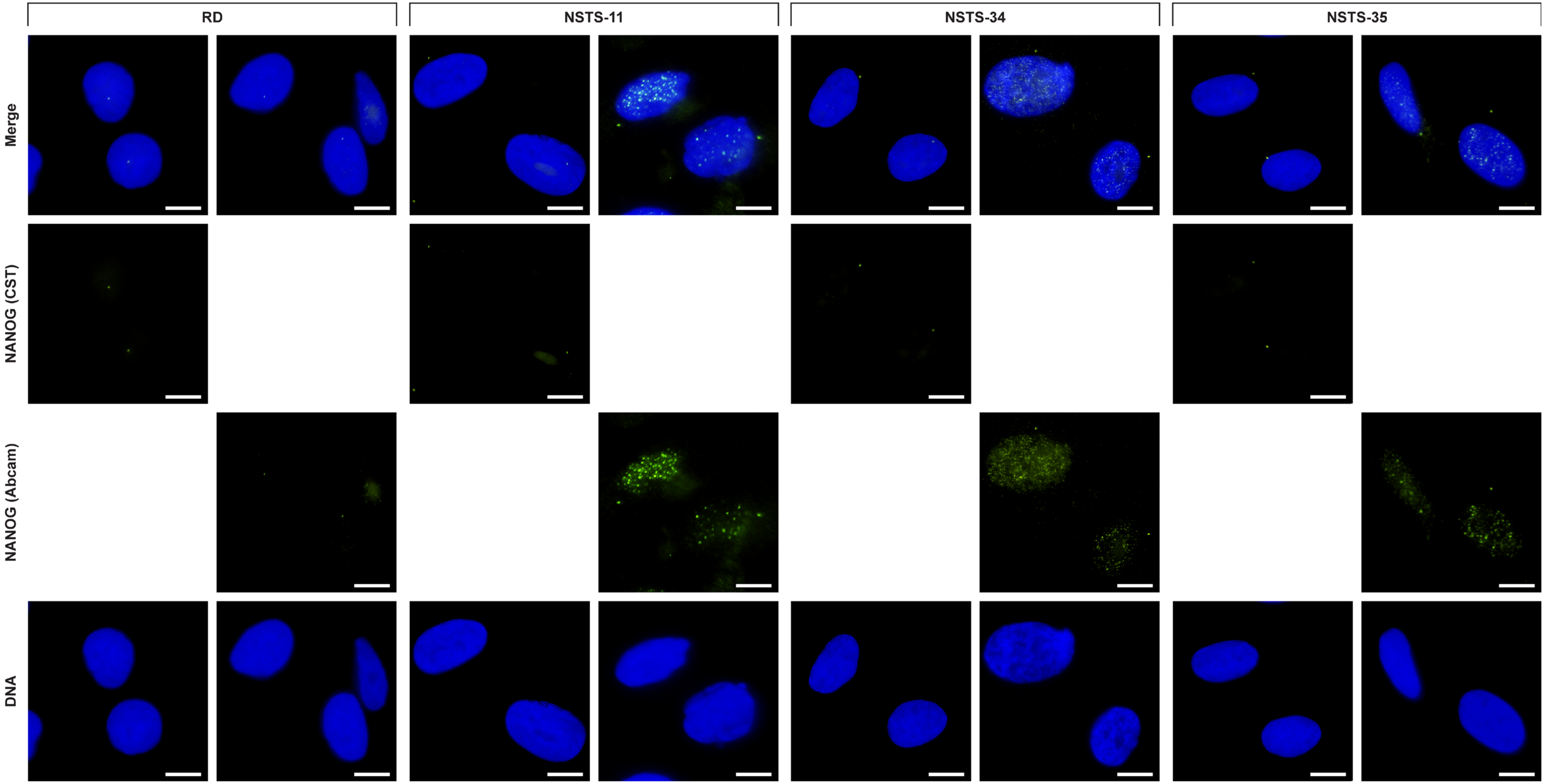

Figure S1. Unmerged images from Figure 1b. Localization of NANOG (green) was detected using two anti-NANOG antibodies (#4903, Cell Signaling Technology [CST] and #ab109250, Abcam). The nuclei were counterstained with Hoechst 33342 (blue). Scale bars, 10 μm.

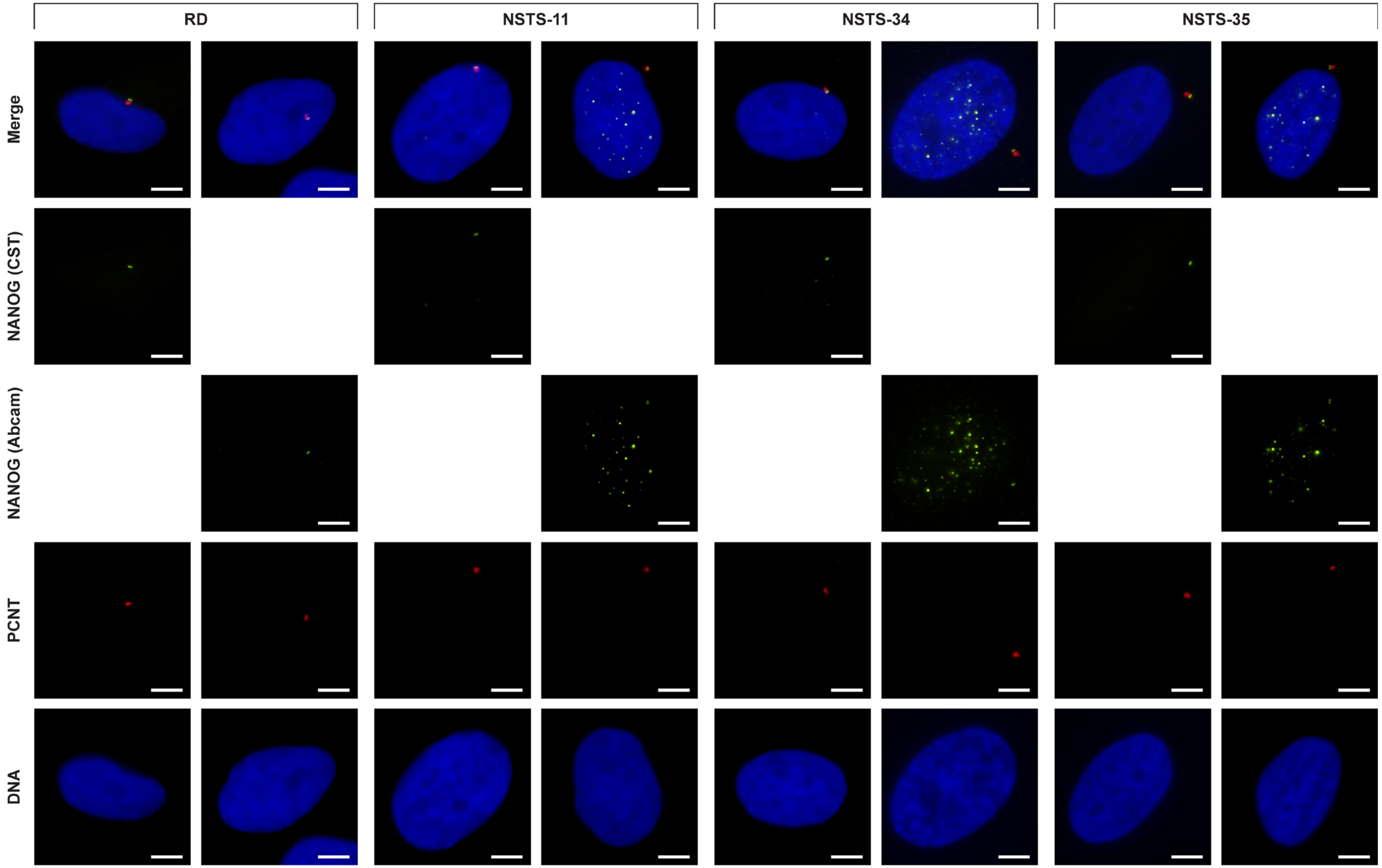

Figure S2. Unmerged images from Figure 3a. Localization of NANOG (green) was detected using two anti-NANOG antibodies (#4903, Cell Signaling Technology [CST] and #ab109250, Abcam) and anti-pericentrin antibody (PCNT; red). The nuclei were counterstained with Hoechst 33342 (blue). Scale bars, 5 μm.

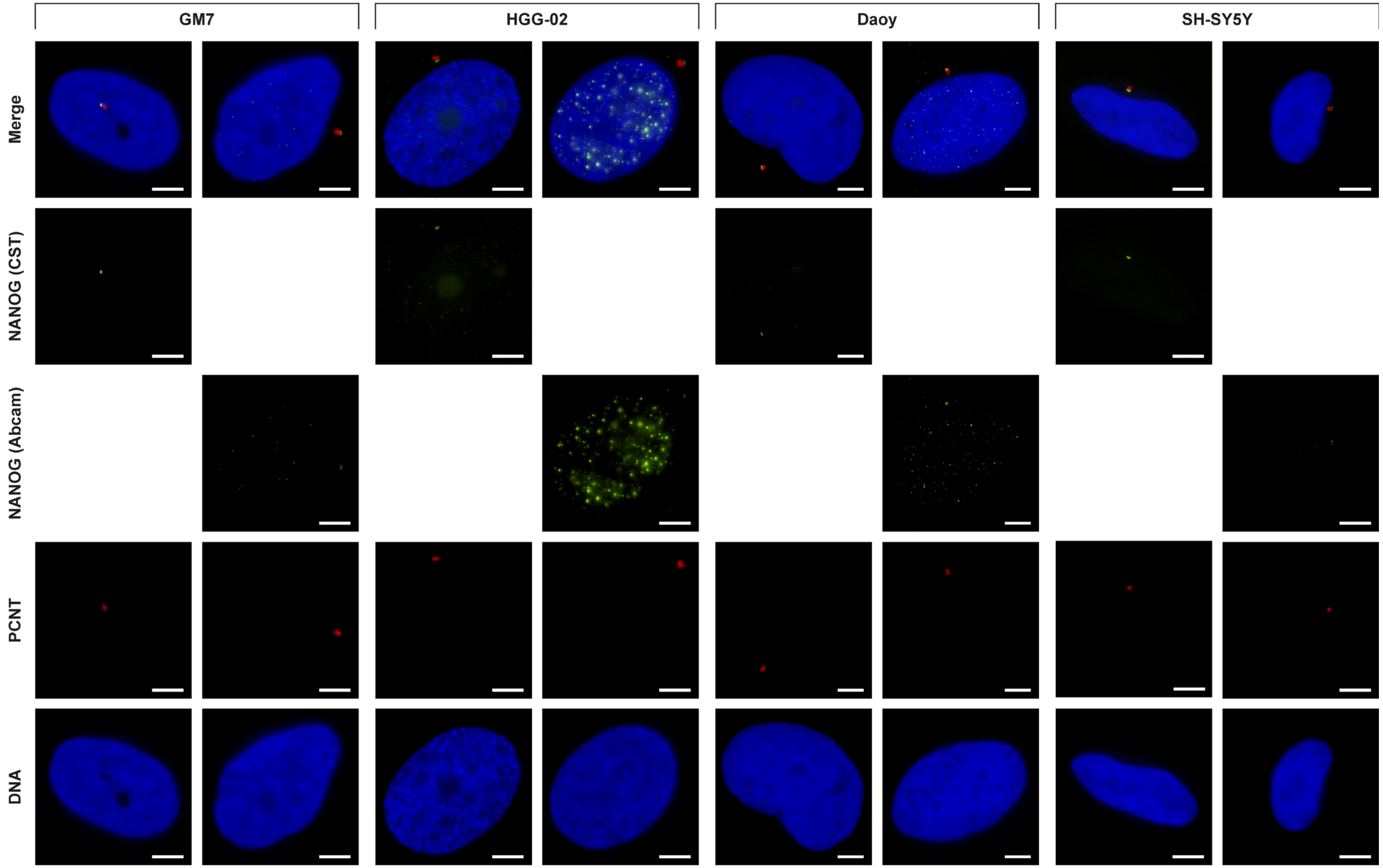

Figure S3. Unmerged images from Figure 3b. Localization of NANOG (green) was detected using two anti-NANOG antibodies (#4903, Cell Signaling Technology [CST] and #ab109250, Abcam) and anti-pericentrin antibody (PCNT; red). The nuclei were counterstained with Hoechst 33342 (blue). Scale bars, 5 μm.

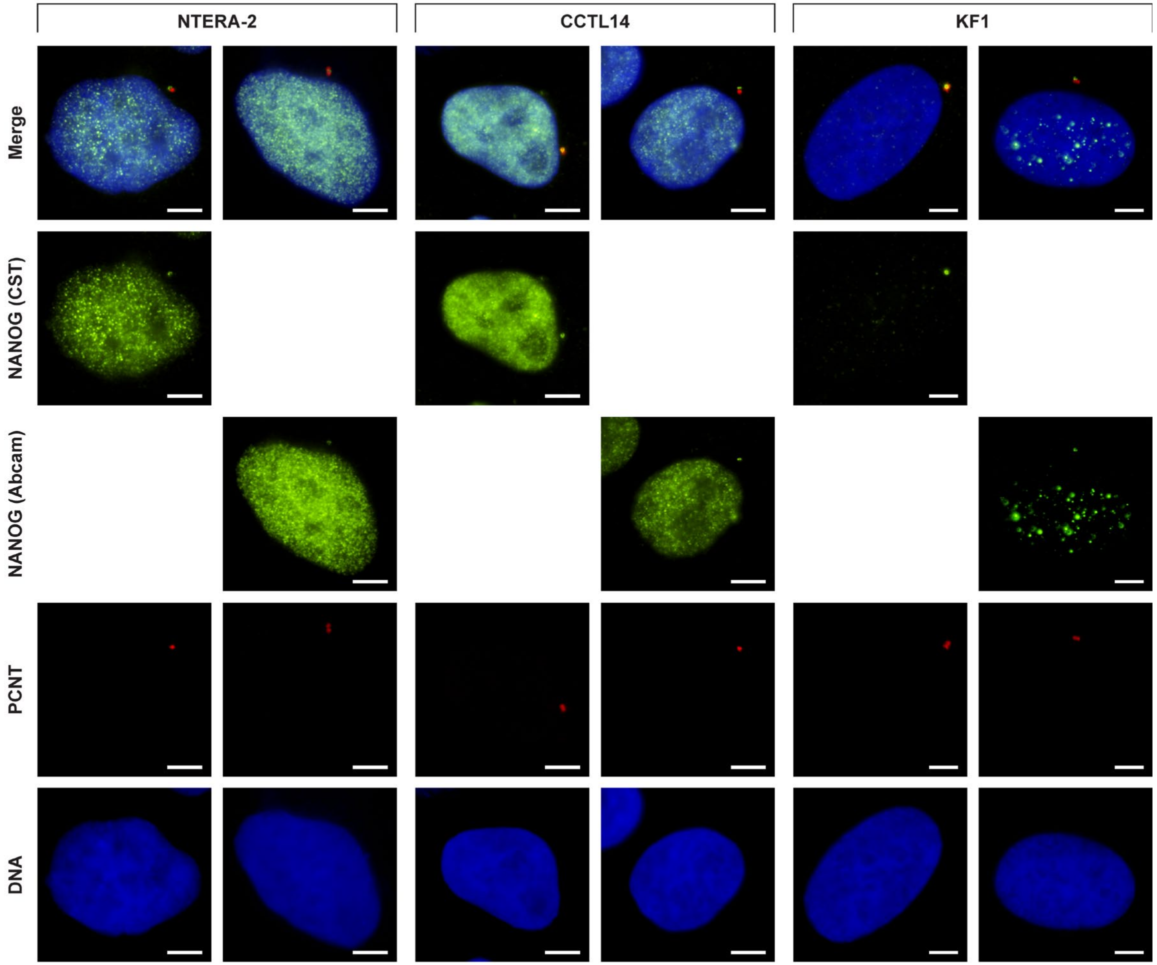

Figure S4. Unmerged images from Figure 3c and Figure 4. Localization of NANOG (green) was detected using two anti-NANOG antibodies (#4903, Cell Signaling Technology [CST] and #ab109250, Abcam) and anti-pericentrin antibody (PCNT; red). The nuclei were counterstained with Hoechst 33342 (blue). Scale bars, 5 μm.

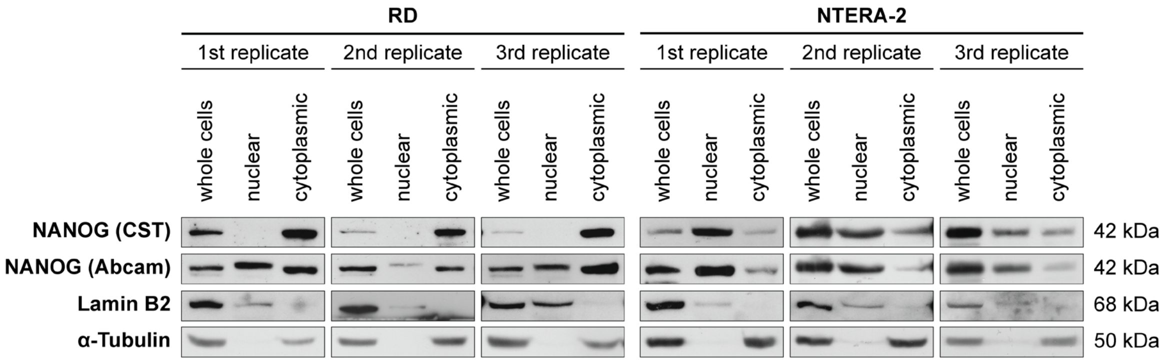

Figure S5. Western blot images of NANOG immunodetection in three biological replicates of the nuclear and cytoplasmic fractions of RD and NTERA-2 cells. Nuclear/cytoplasmic fractionation followed by immunoblotting was performed using two independent anti-NANOG commercial antibodies (#4903, Cell Signaling Technology [CST] and #ab109250, Abcam). Lamin B2 and α-tubulin served as controls of the nuclear fraction and cytoplasmic fraction purity, respectively.

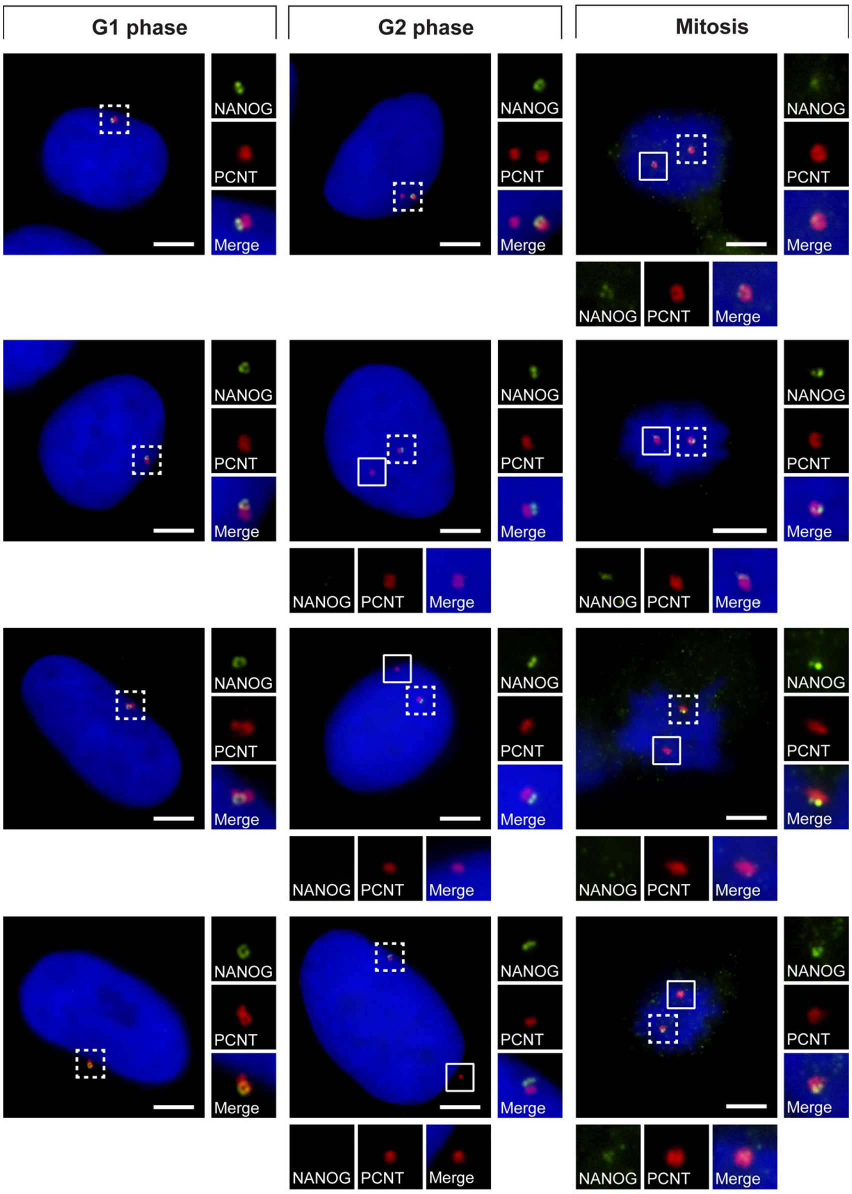

Figure S6. NANOG localization during the cell cycle. NANOG (green; #4903, Cell Signaling Technology [CST]) colocalized with one centrosome (pericentrin, PCNT; red) only during G1 and G2 phases but with both centrosomes (pericentrin, PCNT; red) during mitosis. The nuclei were counterstained with Hoechst 33342 (blue). For each image, regions of interest are indicated by the dashed and solid boxes, and the close-ups are provided on the right and below, respectively. Scale bars, 5 μm.

Table S1. Validation of anti-NANOG #ab109250 antibody specificity by mass spectrometry of immunoprecipitated NTERA-2 cells.

Accession	Gene	Gene symbol	Peptides Count	Spectra Count
P35580	Myosin-10	*MYH10*	45	144
Q13813	Spectrin alpha chain, non-erythrocytic 1	*SPTAN1*	31	70
Q01082	Spectrin beta chain, non-erythrocytic 1	*SPTBN1*	23	51
P35579	Myosin-9	*MYH9*	14	42
Q16643	Drebrin	*DBN1*	13	38
P11142	Heat shock cognate 71 kDa protein	*HSPA8*	11	41
Q8TEW0	Partitioning defective 3 homolog	*PARD3*	10	23
P05787	Keratin, type II cytoskeletal 8	*KRT8*	9	30
P10809	60 kDa heat shock protein, mitochondrial	*HSPD1*	8	20
P14866	Heterogeneous nuclear ribonucleoprotein L	*HNRNPL*	8	43
P25705	ATP synthase subunit alpha, mitochondrial	*ATP5F1A*	8	33
Q9UQ35	Serine/arginine repetitive matrix protein 2	*SRRM2*	8	12
O15164	Transcription intermediary factor 1-alpha	*TRIM24*	7	30
P05783	Keratin, type I cytoskeletal 18	*KRT18*	7	26
O00264	Membrane-associated progesterone receptor component 1	*PGRMC1*	5	13
P04843	Dolichyl-diphosphooligosaccharide--protein glycosyltransferase subunit 1	*RPN1*	5	11
P11021	Endoplasmic reticulum chaperone BiP	*HSPA5*	5	7
P60660	Myosin light polypeptide 6	*MYL6*	5	18
P78527	DNA-dependent protein kinase catalytic subunit	*PRKDC*	5	8
Q00839	Heterogeneous nuclear ribonucleoprotein U	*HNRNPU*	5	8
Q07157	Tight junction protein ZO-1	*TJP1*	5	9
Q92734	Protein TFG	*TFG*	5	22
P01870	Ig gamma chain C region		4	23
P04406	Glyceraldehyde-3-phosphate dehydrogenase	*GAPDH*	4	16
P08670	Vimentin	*VIM*	4	8
P09493	Tropomyosin alpha-1 chain	*TPM1*	4	28
P35221	Catenin alpha-1	*CTNNA1*	4	5
P43243	Matrin-3	*MATR3*	4	5
P61978	Heterogeneous nuclear ribonucleoprotein K	*HNRNPK*	4	8
P62805	Histone H4	*HIST1H4A*	4	32
Q9H444	Charged multivesicular body protein 4b	*CHMP4B*	4	14

Accession	Gene	Gene symbol	Peptides Count	Spectra Count
Q9NYL9	Tropomodulin-3	*TMOD3*	4	5
P05023	Sodium/potassium-transporting ATPase subunit alpha-1	*ATP1A1*	3	5
P23396	40S ribosomal protein S3	*RPS3*	3	9
P35613	Basigin	*BSG*	3	12
P46779	60S ribosomal protein L28	*RPL28*	3	5
P49411	Elongation factor Tu, mitochondrial	*TUFM*	3	7
P52272	Heterogeneous nuclear ribonucleoprotein M	*HNRNPM*	3	7
P56747	Claudin-6	*CLDN6*	3	10
Q07020	60S ribosomal protein L18	*RPL18*	3	9
Q07955	Serine/arginine-rich splicing factor 1	*SRSF1*	3	5
Q15393	Splicing factor 3B subunit 3	*SF3B3*	3	4
Q16629	Serine/arginine-rich splicing factor 7	*SRSF7*	3	13
Q2KHR3	Glutamine and serine-rich protein 1	*QSER1*	3	6
Q5PRF9	Protein Smaug homolog 2	*SAMD4B*	3	4
**Q6NSW7**	**Homeobox protein NANOGP8**	***NANOGP8***	3	6
	GKQPTSAENSVAK (NANOGP8)			
	QPTSAENSVAK (NANOGP8)			
	TVFSSTQLCVLNDR (NANOG, NANOGP1 and NANOGP8)			
O75569	Interferon-inducible double-stranded RNA-dependent protein kinase activator A	*PRKRA*	2	3
P04792	Heat shock protein beta-1	*HSPB1*	2	8
P06753	Tropomyosin alpha-3 chain	*TPM3*	2	11
P09496	Clathrin light chain A	*CLTA*	2	4
P17302	Gap junction alpha-1 protein	*GJA1*	2	8
P35232	Prohibitin	*PHB*	2	3
P36578	60S ribosomal protein L4	*RPL4*	2	5
P46776	60S ribosomal protein L27a	*RPL27A*	2	7
P46782	40S ribosomal protein S5	*RPS5*	2	5
P63244	Receptor of activated protein C kinase 1	*RACK1*	2	3
P78310	Coxsackievirus and adenovirus receptor	*CXADR*	2	8
Q00796	Sorbitol dehydrogenase	*SORD*	2	8
Q14257	Reticulocalbin-2	*RCN2*	2	5
Q15392	Delta(24)-sterol reductase	*DHCR24*	2	12
Q9BWH2	FUN14 domain-containing protein 2	*FUNDC2*	2	6
